# A review of malignant pleural mesothelioma in a large North East UK pleural centre

**DOI:** 10.1515/pp-2020-0144

**Published:** 2020-12-29

**Authors:** Declan C. Murphy, Alexander Mount, Fiona Starkie, Leah Taylor, Avinash Aujayeb

**Affiliations:** Northumbria HealthCare NHS Foundation Trust, Care of Tracy Groom, Cramlington, Northumberland, UK; Institute of Genetic Medicine, Newcastle University, Newcastle upon Tyne, UK

**Keywords:** epithelioid, mesothelioma, pleural

## Abstract

**Objectives:**

The National Mesothelioma Audit 2020 showed Northumbria to have low rates of histopathological confirmation, treatment and one-year survival rates for malignant pleural mesothelioma (MPM). We hypothesized that an internal analysis over a 10-year period provides valuable insights into presentation, diagnosis, treatment and outcomes.

**Methods:**

A single-centre retrospective case series of all confirmed MPM patients between 1 January 2009 and 31 December 2019 was performed. Demographics, clinical, radiological and histopathological characteristics and outcomes were collected. Statistical analysis was performed using SPSS V26.0.

**Results:**

A total of 247 patients had MPM. About 86% were male, mean age 75.7 years. Dyspnoea (77.4%) and chest pain (38.5%) were commonest symptoms. 64.9 and 71.4% had pleural thickening and effusion, respectively. About 86.8% had at least one attempt to obtain a tissue biopsy, but histopathological confirmation in only 108 (43.7%). About 66.3% with PS 0 and 1 (62.7% of total cohort) had at least one anti-cancer therapy. Death within 12 months was associated with disease progression within 6 months (p≤0.001). Chemotherapy (p≤0.001) and epithelioid histological subtype (p=0.01) were protective.

**Conclusions:**

This study confirms known epidemiology of MPM, demonstrates variability in practices and highlights how some NMA recommendations are not met. This provides the incentive for a regional mesothelioma multi-disciplinary meeting.

## Introduction

Malignant mesothelioma is an uncommon cancer that can develop in the pleura or the peritoneum [1]. Exposure to asbestos is the major cause of malignant pleural mesothelioma (MPM) and accounts up to 85% of the cases in the United Kingdom (UK) [1]. The use of asbestos products was banned in the UK in 1999 but the latency period between first exposure and subsequent development of disease is approximately 30–40 years. Hence, currently, the UK is most likely at its peak incidence for MPM [2].

The British Thoracic Society [3] and the European Respiratory Society [4] have published guidance to inform the investigation, diagnosis and management of MPM. MPM is more common in men and in the right hemithorax. It often presents with pain and breathlessness secondary to pleural effusions. A wide range of diagnostic investigations are available, from cytological analysis of pleural effusions to image-guided or thoracoscopic pleural biopsies [1], [2], [3], [4]. Management can include chemotherapy, radiotherapy and more recently immunotherapy, in conjunction with supportive measures to alleviate symptoms. Large retrospective studies have shown that advancing age, male sex, advanced stage and non-epithelioid histology are poor prognostic markers [2], [3].

Outcomes of 8,740 patients with MPM derived from the National Lung Cancer Audit from 2008 to 2012 showed significant variation in the care received and outcomes across the UK [5]. A subset of the lung cancer audit, the National Mesothelioma Audit (NMA) is commissioned and funded by the charity Mesothelioma UK and strives to raise the profile of MPM, highlight variability in care and improve patient outcomes [6].

Northumbria Healthcare NHS Foundation Trust (NHCT) has a well-established pleural and cancer service across a large geographical region with a population of roughly 500,000 [7]. There is access to local anaesthetic thoracoscopy (LAT) and indwelling pleural catheter (IPC) insertion, a Mesothelioma UK nurse specialist and a pleural fellow. Our higher incidence of MPM is a direct consequence of the local shipbuilding and construction industry in the early- and mid-twentieth century which used asbestos widely as a building and lagging material [8]. The workforce was predominantly male.

The 2020 NMA report, based on audit data from 2016 to 2018, showed that NHCT performed poorly in histopathological confirmation of tissue samples, treatment rates and survival rates at 12 months after the initial diagnosis [6].

We hypothesized that an internal analysis of all MPM cases in NHCT over a ten-year period would suggest alternative findings to the NMA and provide valuable insights into local processes of presenting characteristics, diagnoses, treatments and outcomes.

## Materials and methods

Local Caldicott approval (reference RPI-1278) was obtained for a retrospective audit of all MPM patients in the Somerset Cancer Register between 1 January 2009 and 31 December 2019. Demographics, clinical, radiological and histological characteristics and outcomes were collected.

### Aims and objectives

We aimed to:

1) Review the clinical presentations, investigations, diagnostics, treatment and outcomes for all MPM patients presenting to our institution over a 10-year period
2) Compare our findings with the published NMA 2020 audit
3) Investigate patient-specific factors and their associations with diagnosis, treatment and outcomes
4) Investigate which variables are significant predictors of mortality within 12 months for MPM patients

### Statistical analysis

Statistical analysis was performed using IBM SPSS V26.0. Continuous variables are presented as mean (±standard deviation) and categorical variables as percentages where appropriate. Chi-squared tests compared categorical variables. Independent-samples *t*-test compared continuous variables. Multiple regression was performed to determine variables which predict mortality within 12 months following diagnosis. A p-value ≤0.05 defined statistical significance.

This study was performed in accordance with the Strengthening the Reporting of Observational Studies in Epidemiology (STROBE) Statement [9] and abides by the principles of the declaration of Helsinki [10].

## Results

### Description of cohort

A total of 247 patients with MPM were eligible for inclusion. Patient characteristics are described in Table 1.

**Table 1:** Baseline characteristics.

Variable	Result
**Age in years** (mean; ±SD; range)	75.7; 8.2; 54–95
**Gender** (n (%)) M; F	221 (86); 36 (14)
**Ethnicity** White British (%)	257 (100)
**Smoking status** (n (%))	21 (10.5); 103 (51.5); 76 (38)
n=200	
Current smoker; previous smoker; never	
**Other respiratory comorbidity** (n (%)) n=234	
**ECOG score at presentation** (n=174)	40 (15.6)
ECOG 0	46 (26.1)
ECOG 1	64 (36.6)
ECOG 2	32 (18.3)
ECOG 3	31 (17.8)
ECOG 4	3 (1.7)
**Presenting symptom**s **(n (%))**	
Chest pain	90 (38.5)
Dyspnoea	182 (77.4)
Weight loss (self-reported)	67 (29.9)
Fatigue	23 (10.1)
Fever	5 (2.2)
Haemoptysis	5 (2.2)
Asymptomatic	17 (7.3)
**Referral route (n (%))**	
Incidental finding in hospital with no symptoms	25 (9.7)
Diagnosis in hospital presenting with mesothelioma symptoms	48 (18.7)
Referral from GP/outpatient department due to incidental finding suggesting possible mesothelioma	32 (12.5)
Referral from GP or ambulatory care presenting with mesothelioma symptoms	135 (52.5)
Route unknown	17 (6.6)
**Staging recorded (%)**	75.2

Mean age was 75.7 years (range: 54–95), 86% were male and all self-reported their ethnicity as White-British. Half of patients were previous smokers and 16% had another confirmed clinically significant pre-existing respiratory condition, most commonly chronic obstructive pulmonary disease in 67% of patients. Asbestosis, pulmonary fibrosis and asthma accounted for the remaining cases.

A total of 52.5% of patients were referred from primary care or via the local ambulatory care service with symptoms suggestive of mesothelioma. Eastern Cooperative Oncology Group (ECOG) performance status was predominantly 0 or 1 (61.6%). The route of admission did not significantly influence the time required to reach a diagnosis.

Common presenting symptoms were dyspnoea (77.4%), chest pain (38.5%), unintentional weight loss (29.9%) and fatigue (10.1%). 7.3% were asymptomatic.

### Imaging

At presentation, 93.8% patients underwent chest radiography. All underwent computed tomography (CT) of the thorax. In 95% of images, a radiological abnormality consistent with mesothelioma was identified. Specific radiological abnormalities are displayed in Table 2.

**Table 2:** Radiological findings.

Variable	Result
**Chest X-ray (n=241)**	
CXR performed	241 (93.8)
Chest X-ray findings (n (%))	230 (95.4)
Abnormality: Right; left; bilateral; info unavailable	136; 83; 9; 2
Pleural plaques	39 (6.2)
Pleural effusion	185 (78.4)
Lung fibrosis	5 (2.1)
Reduced volume	6 (2.6)
Rib destruction	3 (1.3)
Mediastinal lymphadenopathy	3 (1.3)
**CT scan** (**n=245)**	
CT performed	245 (100)
Abnormal findings on CT	233 (95.1)
Abnormality: Right; left; bilateral	133; 85; 15
Pleural plaques	64 (27.2)
Pleural effusion	167 (71.4)
Lung fibrosis	14 (6.1)
Pleural thickening	153 (64.9)
Reduced volume	8 (3.4)
Rib destruction	9 (3.8)
Nodule	90 (43.7)
Mediastinal lymphadenopathy	36 (15.4)

Most commonly, unilateral right-sided abnormalities were observed. On CT, 133 abnormalities were observed only on the right side, compared with 85 on the left side and 15 bilaterally.

The most common CT radiological abnormalities were pleural effusion (71.4%), followed by pleural thickening (64.9%), nodules (43.7%), pleural plaques (27.2%) and mediastinal lymphadenopathy (15.4%)

### Histopathology

Totally, 223 (86.8%) of patients underwent at least one biopsy method for histopathological characterization. The most commonly performed method was a pleural tap (59.1%), followed by LAT (48.6%), CT-guided biopsy (20.2%), ultrasound-guided biopsy (8.9%) and surgical biopsy (4.3%). In 34 (13.2%) patients, no biopsy was performed.

When tissue was obtained using USS-guided biopsy, 90.5% showed a positive finding. This is compared with 87.8% when obtained using CT-guided biopsy, 23% from a pleural fluid cytology and 92% when obtained by a medical thoracoscopy. Surgical biopsy showed 100% positive cytopathological findings.

Thirty-four individuals did not undergo any biopsy. About 5.8% had an ECOG status of 4, 44.4% an ECOG status of 3, 33.3% ECOG status of 2, 2.6% an ECOG status 1 and 11.1% an ECOG status of 0, 8.6% (n=3) had no ECOG status recorded.

Of the total cohort (N-257), 174 (67.7%) showed positive histopathological findings following at least one tissue biopsy method. However, in only 108 cases (43.7%) could the histopathological analysis be used to confirm the MPM subtype; the commonest was the epithelioid subtype (69) then sarcomatoid subtype (18) followed by biphasic subtype (17) and 4 were of another subtypes (3 desmoplastic and 1 deciduoid). The remaining 32.3% showed inconclusive findings. A diagnosis of an epithelioid subtype of mesothelioma was not significantly associated with smoking status, ECOG status, time to diagnosis or symptoms at their initial presentation (p≥0.05 for all).

### Treatment

Initially, 53.2% of patients with MPM were observed. Of those, 42.9% received chemotherapy for their first treatment intervention, and 15.6% underwent radiotherapy. Rarely, patients received immunotherapy (2) or surgery (1). Of those who were initially observed, one patient underwent surgery as part of a trial when disease progression was noted within 6 months after the initial diagnosis. In total, 101 (39.3%) received at least one anti-cancer treatment.

### ECOG status 0–1

A total of 101 participants had an ECOG status or 0–1. Mean age was 74.12 years (range: 54–92; SD=±7.87) and 88 (87.1%) were male. 95% had biopsies for histopathological examination using at least one method; tissue samples provided positive histopathological findings for subtyping in 66.3%.

Sixty-seven (66.3%) individuals underwent at least one type of anti-cancer treatment, and the other 33.7% were observed as an initial intervention. Of those who received an anti-cancer treatment, 68.4% received chemotherapy and 24.2% of radiotherapy. One patient underwent immunotherapy and one surgery.

A total of 51.5% of individuals with an ECOG status of 0–1 died within 12 months after their initial diagnosis. An additional 31.7% died between 1 and 2 years and 9.9% between 2 and 3 years. Four died between 3 and 5 years following their diagnosis, and similarly four after more than five years.

### Disease progression

At five-year follow-up, 16.9 percent showed no clinical or radiological evidence of disease progression. Most commonly (83.6%), disease progression was evident within 12 months following diagnosis (63.3% within 6 months and 20.3% between 6 and 12 months). Epithelioid histopathological subtype was significantly and negatively associated with disease progression within 6 months following diagnosis (OR=0.24, p=0.01). All other variables were not significantly associated with disease progression, including ECOG status.

### Mortality

A total of 240 patients had died at the time of analysis (August 2020). About 65.9% of patients died within 12 months of their diagnosis, 38.1% within 6 months following diagnosis and 27.8% between 6 and 12 months.

Multiple regression was used to predict death within 12 months after their diagnosis. Variables relating to baseline characteristics, time to diagnosis, treatments, histopathological subtype and disease progression were inserted into the model in a step-wise fashion. A number of variables were independently associated with death within 12 months, including epithelioid subtype, individuals observed or who received chemotherapy as a first intervention and those with evidence of disease progression within 6 months (p≤0.05 for all). Once variables were adjusted for each other, disease progression within 6 months (beta=0.54, p≤0.001), chemotherapy (beta=−0.41, p≤0.001) and epithelioid histological subtype (beta=−0.31, p=0.01) were significant predictors of death within 12 months after diagnosis (R^2^=0.61, p≤0.001).

## Discussion

Our large retrospective case series audit provides valuable insights about current processes in our institution.

Our epidemiological data are in line with the published literature that MPM is more common in elderly men with respiratory co-morbidities and in the right hemithorax. The commonest symptoms at presentation were dyspnoea and chest pain. Common radiological findings were pleural thickening and pleural effusions, in keeping with published evidence [3], [5]. We also demonstrated that an epithelioid histopathological subtype (beta=−0.31, p=0.01) and receiving chemotherapy as a first-line therapy (beta=−0.41, p≤0.001) are protective factors and influence death within-12 months of diagnosis. In contrast, disease progression within 6 months is associated with an increased risk of death within 12 months (beta=0.54, p≤0.001). In those with epithelioid subtypes, disease progression within 6 months of diagnosis is less likely to occur compared within non-epithelioid subtypes (OR=0.24, p=0.01).

National mesothelioma audits were published in 2014 and 2016 for the 2008–2012 and 2014 cohorts, respectively [6]. However, specific data for our trust were not made available from the published summary sheets on their website [6]. The NMA report for 2018 and 2020 covered audit periods of 2014–2016 and 2016–2018, respectively. The NMA audit sets out recommendations in their final report [6]. Comparisons between our study and these cohorts are shown in Table 3.

**Table 3:** comparison between NMA audits and current study.

	Period covered	Patient numbers	ECOG PS recorded (%)	Staging recorded (%)	Pathological confirmation (%)	Anti-cancer treatment (%)	Survival at 1 year (%)
NMA 2018 report	2014–2016	118	64.4	74.6	78.8	48.3	39.1
NMA 2020 report	2016–2018	92	90.2	80.4	77.2	39.1	37
Current study	2009–2019	247	70.4	75.2	54.1	39.3	34.1

We failed to achieve adequate data completeness for ECOG performance status (PS) and staging of disease, for which NMA suggest ≥90% should have this information recorded. The local lung cancer leads and mesothelioma nurse have been informed, as well as the local radiologist to try improving the documentation of those parameters. We have a clinical data lead who will also encourage staff and set targets for staging and PS documentation and all cases are discussed in a multi-disciplinary team (MDT) meeting. This is surprising as the local MDT sheet already has a box for PS (Annex 1). A snapshot review of 20 notes of patients whose PS had not been recorded showed that none of the clinic letters or documentation had a PS recorded and none of those clinicians attended the MDT. This may explain the poor recording of information which has been reported to the 14 respiratory consultants in the trust.

We did not measure if a cancer specialist nurse is present at all consultations (recommended for ≥80% of cases) and if signposting to MesoUK resources is performed (recommended for ≥80% of cases). Further internal analysis is ongoing in that respect.

All patients with adequate PS 0–1 should be offered active anti-cancer treatment and 60% or above of patients with PS 0–1 need to be referred for chemotherapy. At our institution, 95% of patients with PS 0–1 had a biopsy of some sort, and 66.3% were referred for at least one type of anti-cancer treatment, with 68.4% received chemotherapy and 24.2% radiotherapy. This is above the percentage recommendations. However, overall rates of anti-cancer treatments are low. Patient and clinician choice might have a part to play. The latter might be due to an ingrained nihilism when treating mesothelioma patients which was described by Beckett et al. [5] and confirmed by Warby et al. where clinical nihilism in patient care approached 70% [11]. Therapeutic or clinical nihilism in medicine is a concept that arose in the 19th century that treatments such as systemic chemotherapy can do more harm than good. However, nihilism is a clear barrier to evidence-based care and underfunding for research [12]. Bibby et al. prospectively studied the characteristics of mesothelioma patients choosing active symptom control over chemotherapy as first-line treatment [13]. Patients were concerned with side effects, modest survival benefit conferred by chemotherapy and previous adverse experiences with chemotherapy and thus chose active symptom control. For our study, the notes of 10 random MPM patients were pulled. In 4 cases, the clinician opted for observation and in 6 cases, the patients chose not to have any treatment. No specific reasons were given and the phrase “concern over doing more harm” was noted twice. The retrospective nature of this study does not allow for further in-depth analysis. As Bibby et al. suggest, further prospective, qualitative research might be helpful to elucidate this particular question [13].

Our institution failed to obtain sufficient rates of histopathological confirmation compared with the expected rate of ≥95% recommended by NMA. In over 85% of patients from our institution, attempts to obtain a biopsy for histopathology were made however but the actual rates of histopathological confirmation were substantially lower as described in Table 3. Perhaps this is expected because technologies such as immunohistochemistry stains, including BAP1 testing and p16 by fluorescence in situ hybridization (FISH), have only been available for clinical use relatively recently at our institution and have transformed our ability to successfully confirm a final histopathological diagnosis [3]. Unpublished data recently presented at the European Respiratory Society summarizes local challenges regarding timely processing of histopathological samples. They often have to be sent for external reporting due to the lack of specific thoracic pathologists and inability to perform detailed immunohistochemistry for mesothelioma diagnosis [3], [4], [11]. About 34 of our patients did not undergo any type of biopsy, and 4 of those had a PS of 0 and 1 due to patient and clinician choice.

Our institution forms part of the Northern Cancer Alliance and does not have access to regional mesothelioma MDT. The UK Department of Health’s 2007 Mesothelioma Service Framework advised on specialist mesothelioma MDT meetings [13]. This is endorsed in the updated BTS guidance [3]. Mesothelioma MDTs have been shown proven to improve rates of staging, diagnosis, classification by subtype and treatment by concentrating histopathological and clinical-radiological expertise [3], [14], [15]. This will be achievable by increasing access to diagnostic techniques such as medical thoracoscopy (associated with very high diagnostic sensitivity) which is only offered in two hospitals regionally and having expert cardiothoracic pathologists review any samples which will increase the rate of confirmation in a timely fashion. Unpublished data from our centre (presented at the European Respiratory Society Meeting 2020) showed that the length of time for external reporting of pathological samples is 44 days [16]. Mesothelioma MDTs also obviate the need for post-mortem examination [15]. Ahmed et al. found documentation of PS status, treatment rates and access to clinical research to be improved with their regional MDT [17]. Northumbria HealthCare also employs the only regional Mesothelioma UK nurse and access to those specific services will be increased if all patients are discussed in one MDT. Barriers to the development of a self-sustaining MDT have been lack of time available in consultant job plans, problems with access to virtual MDT platforms regionally due to the incompatibility of different electronic platforms being used (Microsoft Teams, Star Leaf and Zoom) and the thoughts that local lung cancer MDTs have already been discussing mesothelioma cases for many years and that a regional MDT might dilute the existing knowledge and future experience. However, the above variability in practice and failure to achieve national recommendations provide strong evidence to develop a regional MDT. The Northern Cancer Alliance has been contacted and expressions of interest have been received from the pleural medicine leads in almost every trust, the regional cardiothoracic pathologists and a regional thoracic radiologist. A referral form for the MDT is currently being developed.

### Limitations

Our study has numerous limitations. It is a single-centre retrospective case study and therefore has limited generalizability. We would encourage other UK centres to publish their data and challenges regarding establishing a regional MDT for comparison. No prospective power-calculation was performed so the study may be prone to type II errors.

## Conclusions

This study confirms known epidemiology of MPM, demonstrates variability in practices between institutions and highlights how some NMA recommendations are not being met. We also showed that disease progression within six months is a significant predictor for death within 12 months. Chemotherapy and an epithelioid histological subtype are protective. In this future, a regional mesothelioma multi-disciplinary team meeting should be established to meet NMA recommendations. Comprehensive data should be collected prospectively so the effectiveness of the MDT can be evaluated, and changed if necessary.

## Highlights

– The National Mesothelioma Audit (NMA) shows clear variance in care in the United Kingdom
–The North East of England has high rates of mesothelioma, and clinical parameters are in line with known epidemiology
– Multiple regression showed disease progression within 6 months (p≤0.001), chemotherapy (p≤0.001) and epithelioid histological subtype (p=0.01) were significant predictors of death within 12 months (R^2^=0.61, p≤0.001).
– The set-up of a Regional Mesothelioma MDT will help meet NMA recommendations
